# Correlation of interferons and autoimmune aspects in long COVID-19 patients

**DOI:** 10.1093/intimm/dxaf008

**Published:** 2025-02-08

**Authors:** Fumiyuki Hattori, Junji Nishiyama, Hideaki Hasuo

**Affiliations:** Innovative Regenerative Medicine, Graduate School of Medicine, Kansai Medical University, Shinmachi 2-5-1, Hirakata, Osaka 573-1010, Japan; Department of Psychosomatic Medicine, Kansai Medical University, Shinmachi 2-5-1, Hirakata, Osaka 573-1090, Japan; Department of Psychosomatic Medicine, Kansai Medical University, Shinmachi 2-5-1, Hirakata, Osaka 573-1090, Japan

**Keywords:** autoimmune disease, PASC, SLE

## Abstract

Long COVID, or post-acute sequelae of COVID-19 (PASC), represents a major global health challenge, with its underlying mechanisms remaining poorly understood despite substantial research and clinical trials. This study investigates the role of the interferon (IFN) axis in the pathogenesis of PASC, drawing parallels to systemic lupus erythematosus (SLE). The potential pathogenic role of IFNs was detected by meta-analyses of mRNA sequencing data comparing PASC patients to healthy controls. We analyzed serum samples from 39 PASC patients and found significant correlations among multiple IFN sub types, including IFN alpha-2, beta, gamma, lambda-1, and lambda-2/3. The biological activity of IFNs in the serum was positively correlated with levels of both total and type III IFNs. Notably, we detected the widespread presence of anti-double-stranded DNA (anti-dsDNA) and anti-Smith (anti-Sm) antibodies in these patients, with anti-dsDNA levels showing a strong correlation with IFN activity. On the basis of these findings, we propose a hypothetical autoimmune pathogenesis for PASC highlighting the crucial role of IFN signaling.

## Teaser

Potential roles for interferons in long COVID

## Graphical Abstract

**Graphical Abstract:**
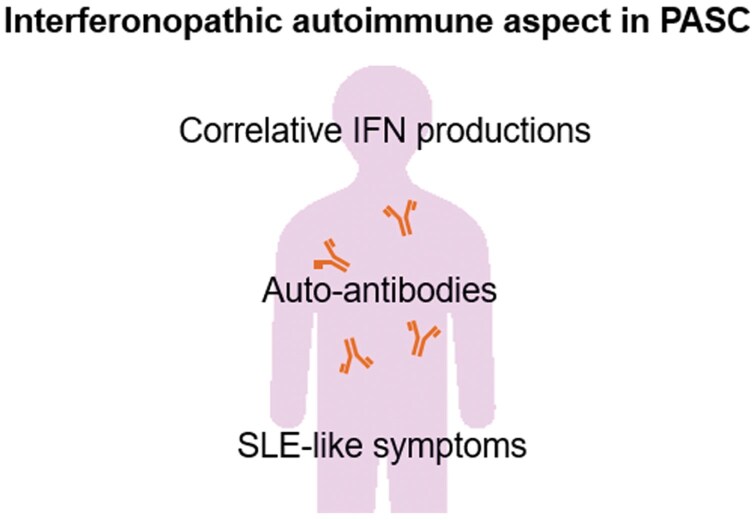

## Introduction

Several pathogenic hypotheses for post-acute sequelae of COVID-19 (PASC) have been suggested, including persisting reservoirs of SARS-CoV-2 in tissues (1); immune dysregulation (2); dysbiosis (3); microvascular and endothelial dysfunction (4); dysfunction of the vagus nerve (5); and autoimmunity (6, 7).

Type I interferonopathy is the pathogenic concept of an autoimmune disease group characterized by excessive type I interferon (IFN) secretion caused genetically and non-genetically, including systemic lupus erythematosus (SLE) (8), rheumatic arthritis, systemic sclerosis, Sjögren’s syndrome, and dermatomyositis. Because of the central pathogenic role of IFN, symptoms of interferonopathies commonly include fever, fatigue, myalgia, and major depressive disorder (9).

We have noted symptomatic similarity between side effects observed in IFN administration therapy and PASC and published a case report describing continuous internal viral shedding and type I IFN activity 1 month after clinical recovery from COVID-19 (10). In this study, we focused on the similarity of symptom profiles of SLE with PASC and investigated the PASC patients’ sera.

## Methods

### Serum sample collection

For the diagnosis of PASC, we adopted the criteria provided by the World Health Organization, which defined PASC as the continuation or development of new symptoms involving fatigue, exhaustion, weakness, dizziness, hair loss, loss of appetite, heart palpitations, depression, anxiety, a high temperature, joint pain, diarrhea, stomachache, cough, headaches, sore throats, and skin rashes 3 months after the initial SARS-CoV-2 infection, with these symptoms lasting for at least 2 months with no other explanation. However, the PASC patients were not diagnosed using the specific blood tests for SLE. The blood samples were obtained from patients on the basis of informed consent. This research was approved by the Ethics Committee on Human Rights Related to Research at Kansai Medical University, Osaka, Japan (No. 2022110).

### Multiplex enzyme-linked immunosorbent assay (ELISA)

We used the LEGENDplex™ Human Type 1/2/3 Interferon Panel with Filter Plate (BioLegend, CA, USA Cat. 740350) to quantify human IFN-α2, IFN-β, IFN-λ1 (IL-29), IFN-λ2/3 (IL-28a/b), and IFN-γ in the serum according to the manufacturers’ instructions. The fluorescent signals were evaluated by an Attune NxT Acoustic Focusing Cytometer with four laser systems (Thermo Fisher Scientific, Waltham, MA, USA). The measurements were conducted within the quantitative range of the assay. For the comparisons between IFN types, we normalized the serum concentration of each IFN divided by their median ratio. The normalized value for each type of IFN was calculated with the averaging of the normalized value(s) of included IFN(s) in it.

### Biological IFN activity

To confirm the biological IFN activity of each sample, we stimulated the human hepatocellular carcinoma cell line, HepG2, with PASC patient serum. HepG2 cells have been used for similar biological assays (10, 11, 12). HepG2 cells were seeded in a 48-well plate with Dulbecco’s modified Eagle’s medium high glucose (Fujifilm Wako Chemical Inc., Osaka, Japan) containing 1× insulin transferrin and selenium mix (Fujifilm Wako Chemical) for 24 hours. The cells were stimulated by patient serum at 10% concentration for 6 hours. Total RNA was recovered from each well by the FastGene RNA Basic Kit (Nippon Gene Co., Ltd., Tokyo, Japan). Of the total RNA, 300 ng of RNA was reverse-transcribed using the SuperScript II cDNA Synthesis Kit (Thermo Fisher Scientific). One microliter of cDNA was amplified with primers (human ISG15 Fw: CACCTGAAGCAGCAAGTGAGCGGGCTGGAG, human ISG15 Rv: CCGCAGGCGCAGATTCATGAACACGGTGCT, human Mx1 Fw: GCCAGCAGCTTCAGAAGGCCATGCTGCAGC, human Mx1 Rv: GGGCAAGCCGGCGCCGAGCCTGCGTCAGCC, human IFIT1 Fw: GCCTTGCTGAAGTGTGGAGGAA, human IFIT1 Rv: ATCCAGGCGATAGGCAGAGATC, human ribosomal protein S18 Fw: GCGGCGGAAAATAGCCTTTG, human ribosomal protein S18 Rv: GATCACACGTTCCACCTCATC) using GeneAce SYBR™ qPCR Mix II (Nippon Gene) and quantitative PCR system, Rotor gene 2 (QIAGEN K.K., Tokyo, Japan). The reaction conditions were initial denaturation at 95°C for 10 minutes, 40 cycles of denaturation at 95°C for 10 seconds, annealing at 57°C for 15 seconds, and extension at 72°C for 20 seconds. To quantify each relative difference, the diluted series of one of the samples with the dilution factor 1:4 was also amplified using each primer pair for developing a standard curve. The threshold cycle, i.e. Cq value, was automatically optimized by the Rotor-Gene Q Software, and the relative expression levels with arbitrary values were calculated based on the standard curve. For normalization of the interest gene expression level, each quantified arbitrary value was divided by the result of human ribosomal protein S18 as an internal control, one of the housekeeping genes (13).

### Anti-double-stranded DNA (anti-dsDNA) and anti-Smith (anti-Sm) ELISAs

The ELISAs for anti-dsDNA anti-Sm antibodies were performed manually following the manufacturers’ instructions (MESACUP DNA-II test and MEBLux test Sm, Medical & Biological Laboratories Co. Ltd., Tokyo, Japan). Colorimetric and luminescent signals were measured by a multi-plate reader (EnSight™, PerkinElmer Japan Co., Ltd., Kanagawa, Japan). The anti-dsDNA antibody assay kit provides the test results obtained from healthy persons (mean value = 5.98 IU/ml, SD = 3.18, maximum value = 19.8 IU/ml, minimum value = 0.00 IU/ml, upper limit by 95% percentile: 12.0 IU/ml). According to the previous report using the same anti-Sm antibody assay kit, the measurement result for healthy controls (*N* = 57) was median 0.5 IU/ml (range 0.1–19.1) (14).

### Meta-analysis of the RNA sequencing data set of PASC

We utilized RNA sequencing data from the Gene Expression Omnibus (GEO) Dataset (GSE251849) (15) involving healthy controls (*N* = 7), recovered from an acute infection of COVID-19 (*N* = 5), and long COVID (general: *N* = 6 and brain fog: *N* = 5), which was processed by the web-based software GREIN. To highlight characteristics of PASC, we constructed a new control group combining healthy controls (*N* = 7) and those recovered from an acute infection of COVID-19 (*N* = 5) and compared two groups: new control (*N* = 12) and PASC (*N* = 11). We extracted the differentially expressed gene (DEG) set by DESeq2 using web software, RNAseqChef. The DEG list (false discovery rate, FDR < 0.05), including 18026 genes (Supplementary Table 1), was processed by SRplot to perform GO- and pathway-enrichment analyses. Among the three major ontologies, biological process, cellular components, molecular function, and pathway enrichment analysis using the Kyoto Encyclopedia of Genes and Genomes (KEGG) were shown.

### Statistical analysis

The outliers were found by the Smirnov-Grubbs test. The identified outliers were included in further analyses. The statistical significance was defined as *P* < .05. Data correlations were analyzed by Spearman’s product-moment correlation and calculated *r* and *P* values. Comparisons in two groups were done by the Student t-test. To exclude potential confounding factors involving gender and age, linear mixed-effects model (LMM) and generalized linear mixed-effects model (GLMM) were applied to the datasets using R version 4.02 with packages of lme4 and glmm handled by EZR version 1.52. To extract possible correlation between symptoms and anti-DNA, anti-Sm antibody titer, and every IFN concentration, multiple regression analyses were applied.

## Results

**Figure 1.**
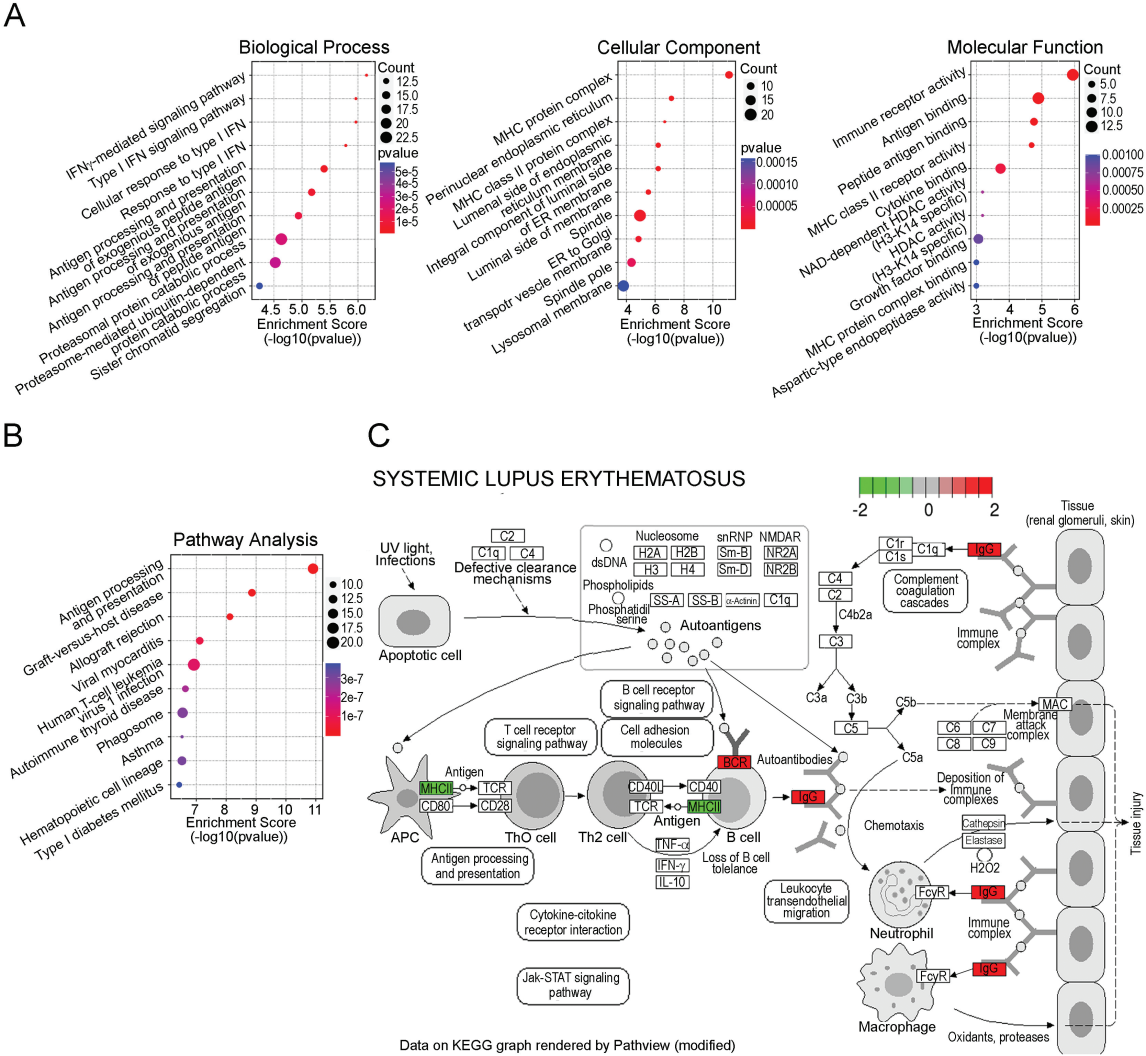
Altered IFN signaling and autoimmune signatures in a meta-analysis of RNA sequencing data of PASC patients’ peripheral blood mononuclear cells. (A) The control group (*N* = 12) combines healthy controls (*N* = 7) and recovered from an acute infection of COVID-19 (*N* = 5). The PASC group (*N* = 11) combines brain fog (*N* = 5) and other PASC (*N* = 6). DESeq2 with RNAseqChef selected the differentially expressed genes (DEGs), which were identified based on *P*-value < .05 without multiple testing corrections. The DEGs, including 18026 genes, were processed by SRplot to perform GO- and pathway-enrichment analyses. The three ontologies: biological process (GO:0008150), cellular component (GO:0005575), and molecular function (GO:0003674) are shown. (B) The result of pathway enrichment analysis using KEGG, and (C) the pathogenic cascade of SLE (16).

For the comprehensive gene expressions comparing PASC with healthy controls, we conducted a meta-analysis of the public RNA sequencing data set using peripheral blood mononuclear cells (PBMCs) deposited by Greene *et al*. (15). To extract the common characteristics of PASC patients, we combined the healthy group and the recovered group from the COVID-19 infection for a new control group data set (*N* = 12), also general PASC patients and brain fog patients for a new PASC group (*N* = 11), and conducted pairwise comparisons. We extracted a significantly DEG set (Supplementary Table 1) and applied it to the gene ontology (GO) enrichment analysis and revealed alterations in type I and II IFN signaling, antigen presentation activity, and antigen processing activities (Fig. 1A). The pathway enrichment analysis revealed that the PBMCs from PASC patients showed autoimmune disease-related signatures (Fig. 1B). Pathway enrichment analysis identified gene expression alterations in MHC class II, IgG, and BCR as components of the SLE pathway (Fig. 1C), although these alone do not adequately represent SLE features.

**Figure 2.**
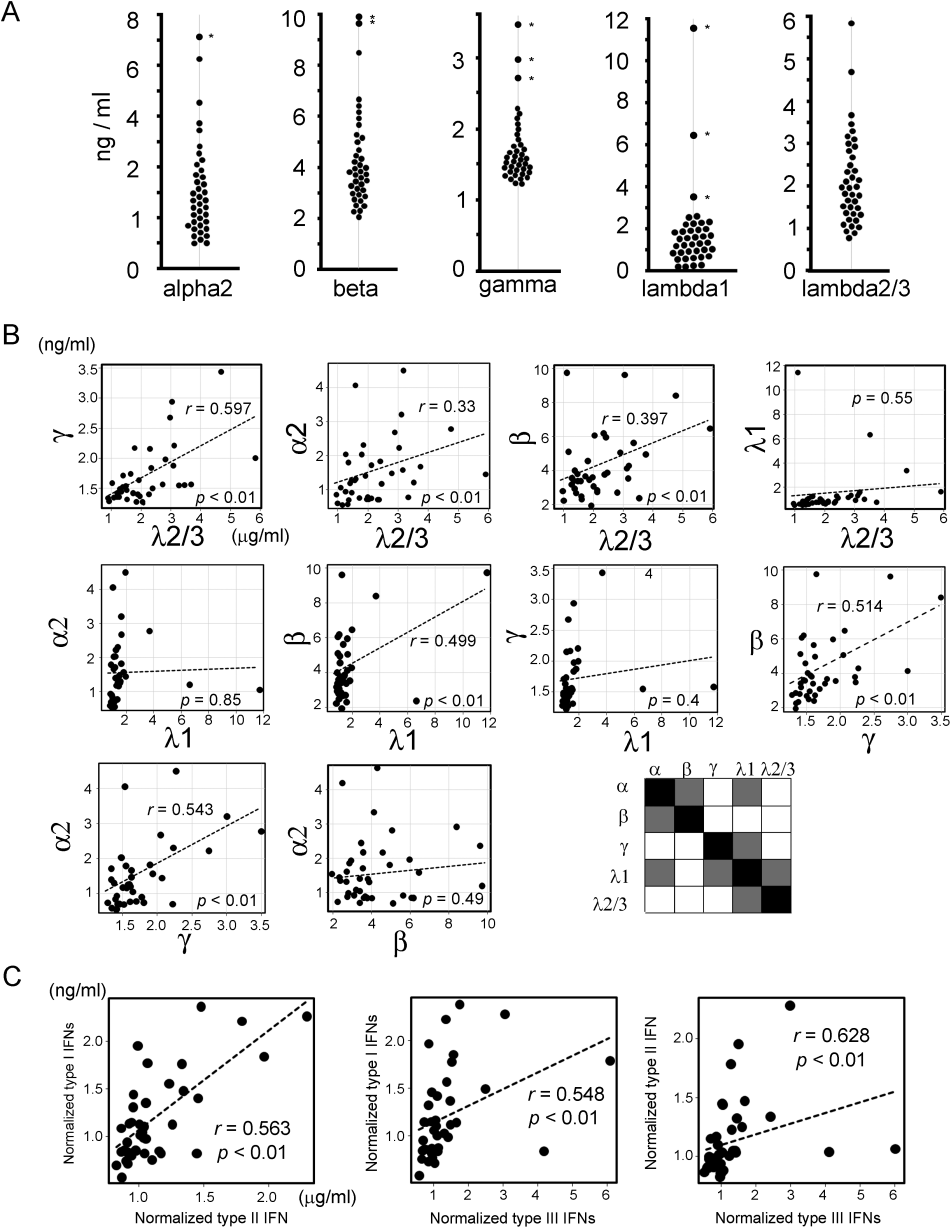
IFN levels and each correlation test in PASC patient serums. (A) Bee swarm plot of IFN levels of PASC patients. Asterisks indicate statistically significant outliners. (B) Correlations of each IFN and (C) each type of normalized IFN were conducted by Spearman’s product-moment correlation and calculated *r* and *P* values. The correlations between IFNs were summarized. White: significant, Gray: not-significant.

We collected serum samples from 39 PASC patients, including 21 women and 18 men. The summary of patients’ information, all data obtained by ELISA, and symptoms is shown in Supplementary Table 2 and Supplementary Fig. 1. Each normal range of IFN was provided by the manufacturer using normal human serum samples (*N* = 18) with the same multiplex ELISA system (Supplementary Table 3), showing lower serum IFN concentrations than those of PASC patients tested here. The obtained data from the multiple ELISA for IFNs includes the statistically significant eight outliers found by the Smirnov-Grubbs test (Fig. 2A). A correlation between some IFNs and different types of normalized IFN levels was detected (Fig. 2B and 2C). No correlations were detected between sex or age and each IFN level by the Spearman’s product-moment correlation. To exclude potential confounding factors involving gender and age, LMM and GLMM were applied (Supplementary Fig. 2). The results suggested possible correlations between all serum IFN concentrations by excluding the effects of gender and age.

**Figure 3.**
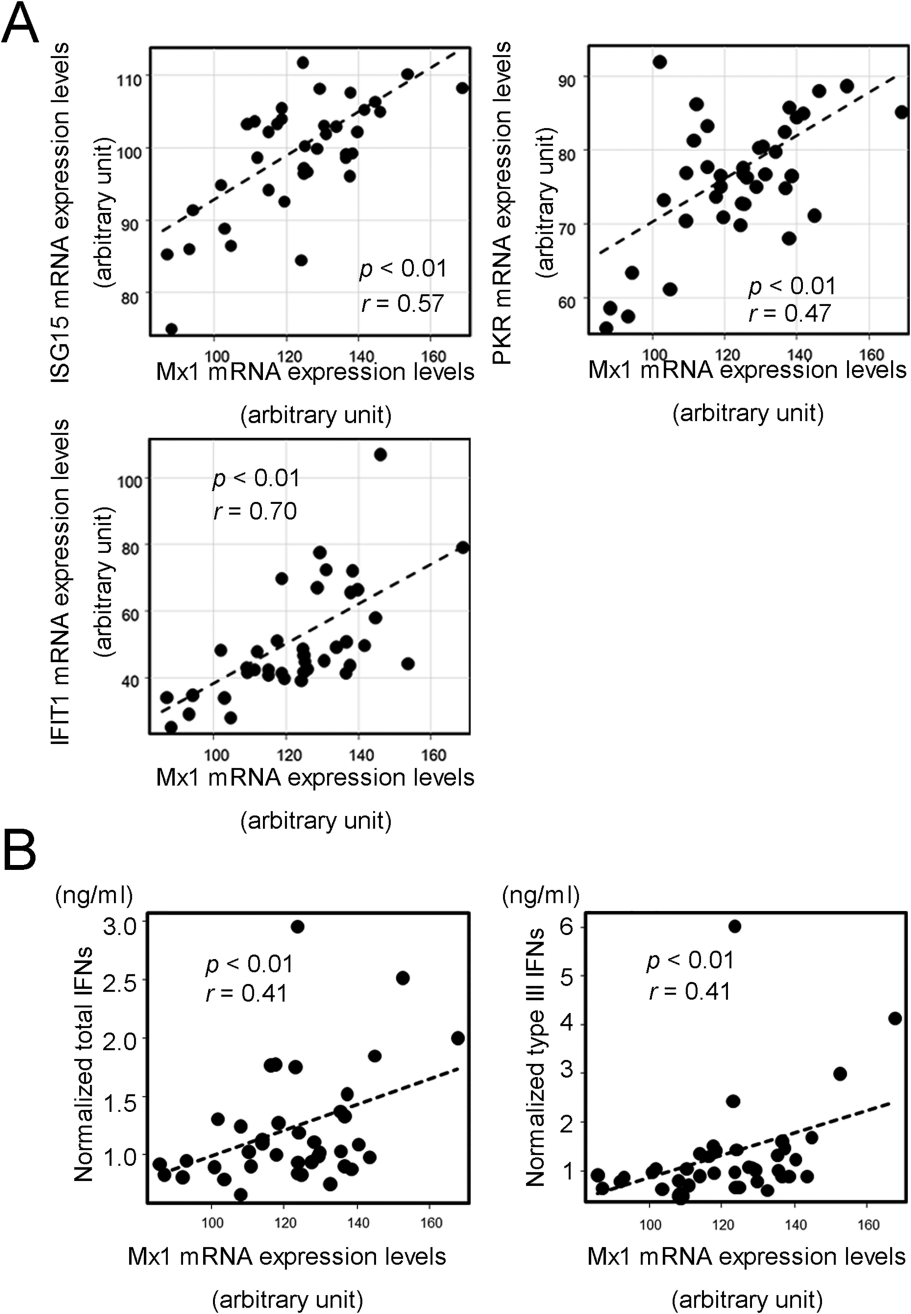
Biological activity of PASC patients’ sera. (A) Positive correlation of IFN activities indicated by Mx1 and PKR, ISG15, or IFIT1 expression levels. (B) Positive correlation of IFN activities indicated by Mx1 expression levels and the normalized total and type III IFN levels. Each mRNA expression level was normalized by that of ribosomal protein S18 as an internal control. Detailed calculation procedures are in the Methods section.

To confirm the biological IFN activity of each sample, we stimulated the human hepatocellular carcinoma cell line, HepG2, with PASC patient serum and investigated the mRNA expression level of IFN-stimulated genes (ISGs) involving Mx1, ISG15, PKR, and IFIT1. The Mx1 gene expression level showed positive correlations with all expression levels of ISGs and normalized total and type III IFN levels of the PASC patient serum (Fig. 3A, B). To exclude potential confounding factors involving gender and age, LMM and GLMM were applied (Supplementary Fig. 3). The results suggested some possible correlations between anti-DNA or anti-Sm antibody titers and many serum IFN concentrations by excluding the effects of gender and age. However, no possible correlation between anti-DNA and anti-Sm antibody levels was detected.

**Figure 4.**
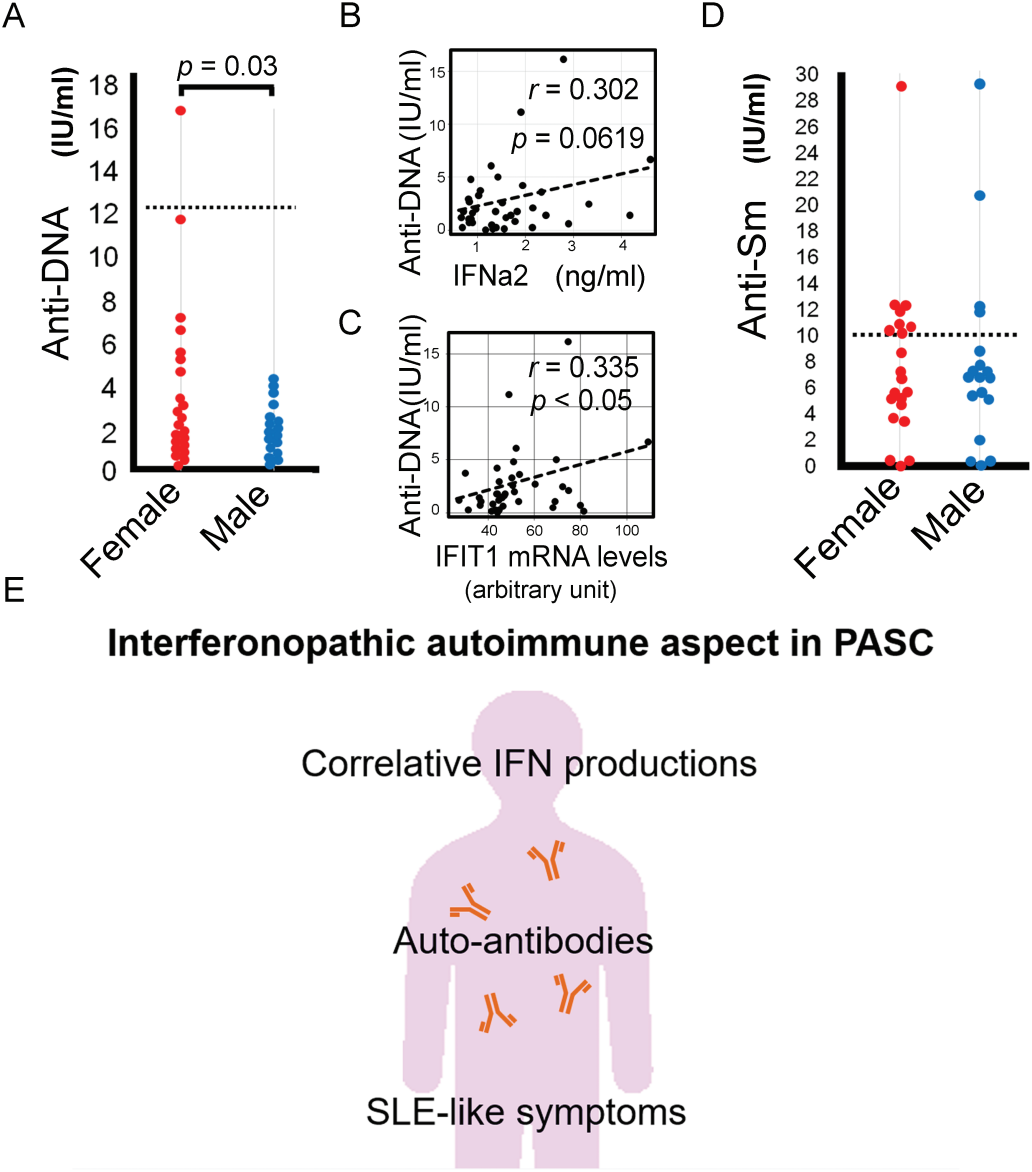
Anti-dsDNA and anti-Sm antibody levels in PASC patient serums. (A) Anti-dsDNA antibody levels of females showed a significantly higher level than males. The dashed line: the diagnostic threshold. (B) Correlation tests of anti-dsDNA antibody levels with IFN α2 or (C) IFIT1 expression levels. The calculated *r* and *P* values were shown. (D) Comparisons of anti-Sm antibody levels between the genders. The dashed line: the diagnostic threshold. (E) Schema of hypothetical pathologies in PASC.

All PASC patients had detectable anti-dsDNA antibody levels; in contrast, a healthy person’s serum had no detectable amount of it. Female showed significantly higher titers than male (Fig. 4A). Only one sample exceeded the clinical diagnostic value for SLE (12 IU/ml). The anti-DNA antibody levels showed a correlative tendency with IFNα2 (Fig. 4B). Furthermore, the IFIT1 gene expression level of the PASC patient-serum stimulated cells showed positive correlations with serum levels of anti-dsDNA antibody (Fig. 4C). Anti-Sm antibody was detected in 33 patients’ serum (84.6%). Among them, eight women (38.1%) and four men (22.2%) have diagnostic positive values for SLE (Fig. 4D). No correlations were detected between anti-Sm antibody levels and each IFN or anti-DNA antibody level by the Spearman’s product-moment correlation. To exclude potential confounding factors involving gender and age, LMM and GLMM were applied (Supplementary Fig. 4). The results suggested some possible correlations between each IFN concentration, anti-DNA or anti-Sm antibody titer and biological activity of serum IFNs by excluding the effects of gender and age. Furthermore, to extract possible correlation between symptoms and anti-DNA, anti-Sm antibody titer, and every IFN concentration, multiple regression analyses were applied. The results were summarized in Supplementary Fig. 5. From our studies, we propose a hypothetical pathogenic model of PASC (Fig. 4E).

## Discussion

In this study, we observed correlative serum levels between all types of IFNs, which is consistent with and complements the previous study showing associations of IFN beta, gamma, and lambda2/3 levels with PASC (17). The correlative elevations of all types of IFNs are rarely informed phenomena in autoimmune diseases. While research has predominantly focused on the elevation of type I IFN, specifically IFN alpha and beta in interferonopathies, there have been fewer investigations into the contributions of type II (18, 19) and type III (20) IFN. Traditionally, the concept of interferonopathy emphasizes the upregulation of type I IFNs and their associated receptor signaling pathways. Our findings of correlated expressions of all IFN types in the serum of PASC patients suggest the involvement of IFNs in the pathogenic feature of PASC.

Continuous elevation of IFN levels is recognized as a key pathogenic driver in SLE (8). In our study, we identified the presence of anti-dsDNA and anti-Sm antibodies in the serum of PASC patients, and the former showed significant correlation with biological IFN activity observed with IFIT1 expression levels. IFIT1 is known as a biological marker gene of SLE (21). The Mx1 expression level in SLE patients’ blood cells is also known to be correlated with disease progression (22, 23). The above finding underscores the pathological similarities between PASC and SLE. While the autoimmune aspects of PASC have been previously suggested (24–26), this study uniquely highlights both the similarities and differences between PASC and SLE, i.e. the presence of anti-dsDNA and anti-Sm antibodies and the coordinated upregulation of all types of IFN, respectively.

Given the similarity of symptoms of side effects of IFN administration and PASC, we previously reported a case showing elevated type I IFN activity in a patient who had continuous internal viral shedding in the saphenous vein one month after clinical recovery from COVID-19 (10). However, the persistent replicative viruses in most chronic PASC patients seem unlikely, as evidenced by the STOP-PASC trial, which did not demonstrate significant benefits from the antiviral drug Paxlovid for PASC (27). Taking them into account, we hypothesize a two-phase model of pathogenesis for PASC. The initial phase may be triggered by residual viral activity, but this can shift to a secondary, long-term autoimmune phase. Both phases are characterized by and may be driven by sustained production of IFNs, which may possibly explain the overlap in symptoms between PASC and various interferonopathies.

The limitation of this study is the lack of a healthy control group. The limitation was partially compensated by the meta-analysis of the public RNA sequencing data set, including healthy and recovered (non-PASC) persons versus PASC patients. The result elucidated the GO and pathway enrichment analyses whose results supported our hypothesis from molecular levels. This study provides a hypothetical pathological model of PASC, which emphasizes the requirement of further studies.

## Supplementary data

Supplementary data are available at *International Immunology* Online.

dxaf008_suppl_Supplementary_Figure_S1-S5

dxaf008_suppl_Supplementary_Table_S1

dxaf008_suppl_Supplementary_Table_S2

dxaf008_suppl_Supplementary_Table_S3
