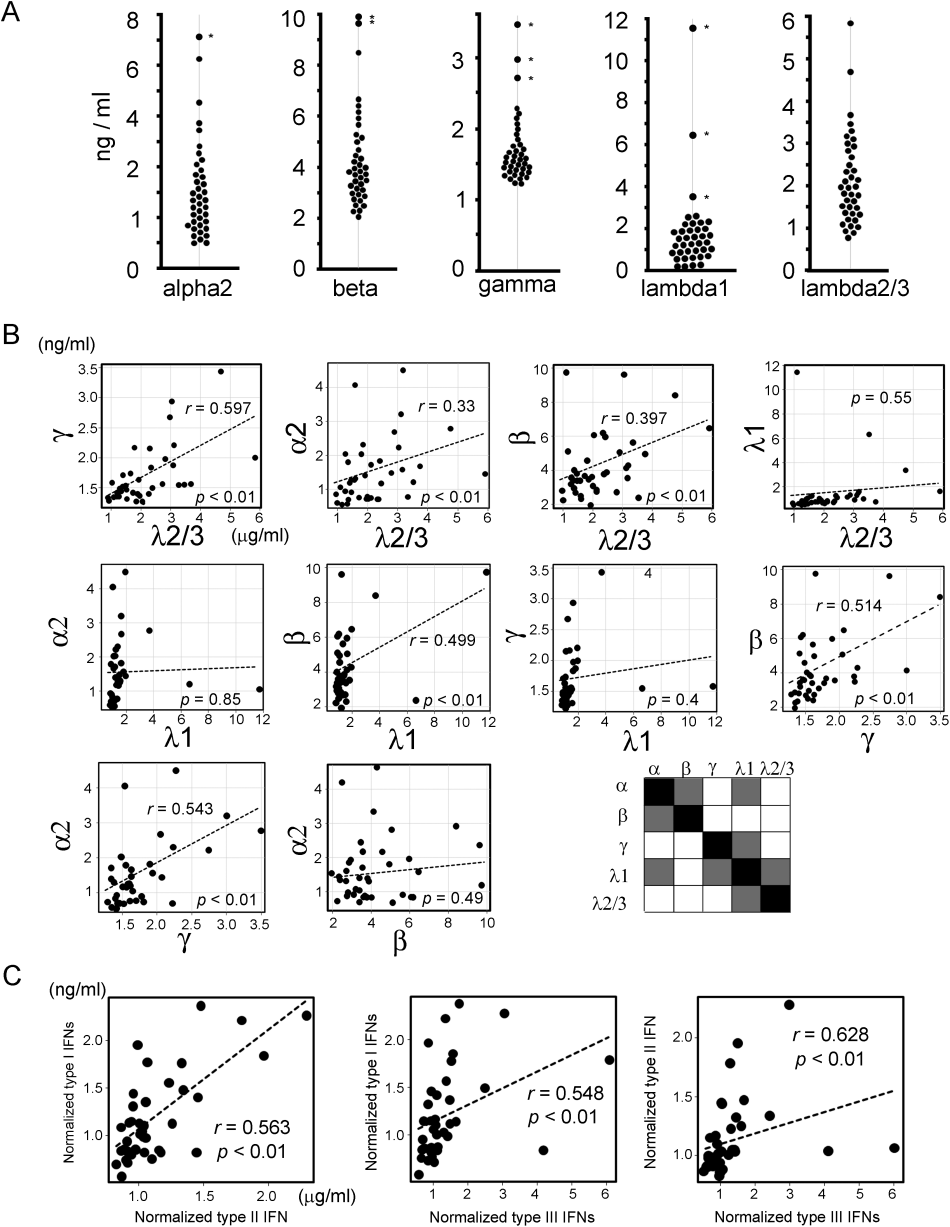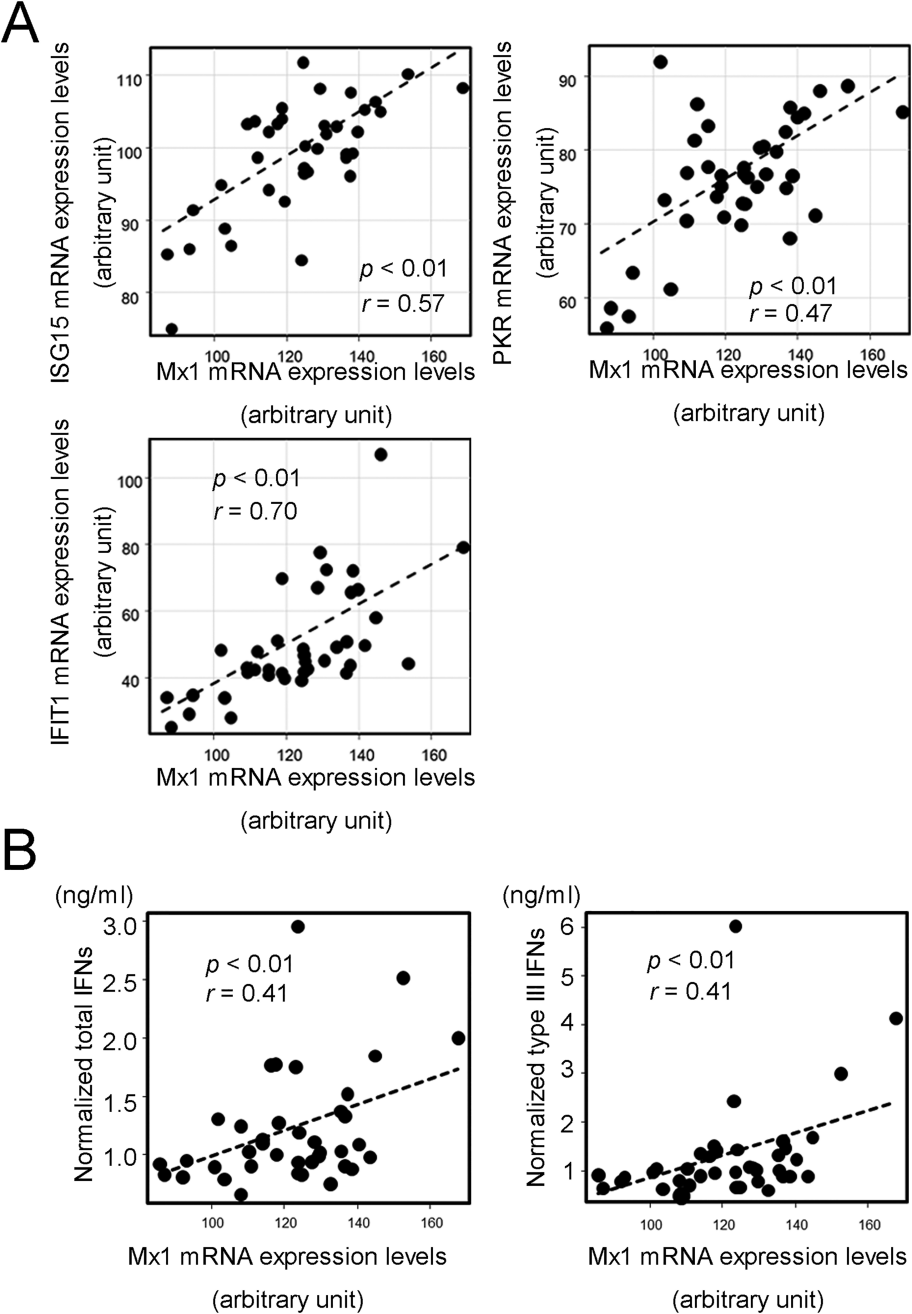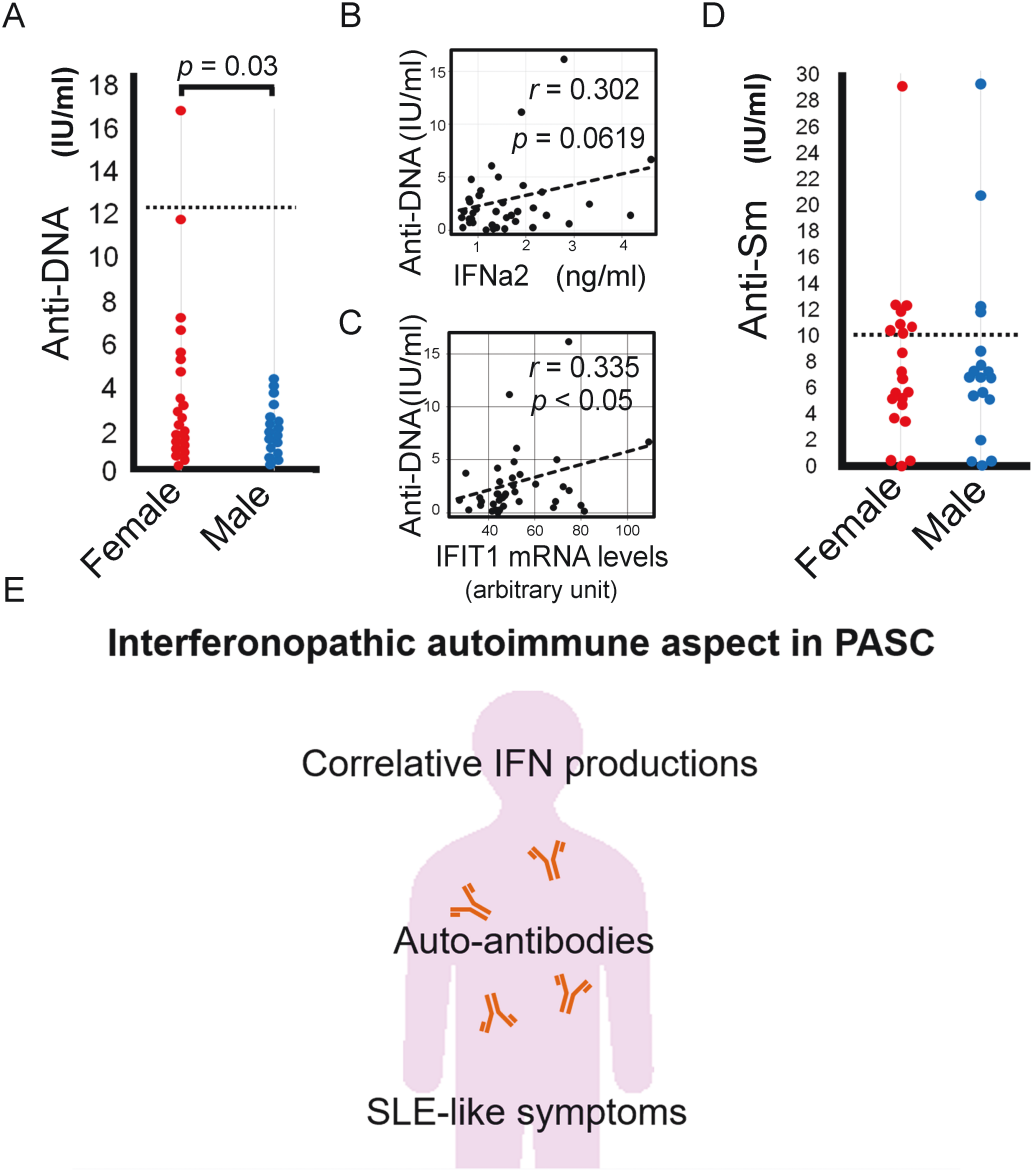# Correction to: Correlation of interferons and autoimmune aspects in long COVID-19 patients

**DOI:** 10.1093/intimm/dxaf044

**Published:** 2025-08-12

This is a correction to: Fumiyuki Hattori, Junji Nishiyama, Hideaki Hasuo, Correlation of interferons and autoimmune aspects in long COVID-19 patients, *International Immunology*, Volume 37, Issue 6, June 2025, Pages 355–363, https://doi.org/10.1093/intimm/dxaf008

Figures 2, 3 and 4 have been updated to correct a typographical error in the units expressed.