# Supplementary material for: Correlation of interferons and autoimmune aspects in long COVID-19 patients
Source: Int Immunol. 2025 Feb 8;37(6):355–63. doi: 10.1093/intimm/dxaf008 (PMC12096164; doi:10.1093/intimm/dxaf008)
Supplement: dxaf008_suppl_Supplementary_Figure_S1-S5 [file dxaf008_suppl_supplementary_figure_s1-s5.pdf]

Supplementary Figure 1

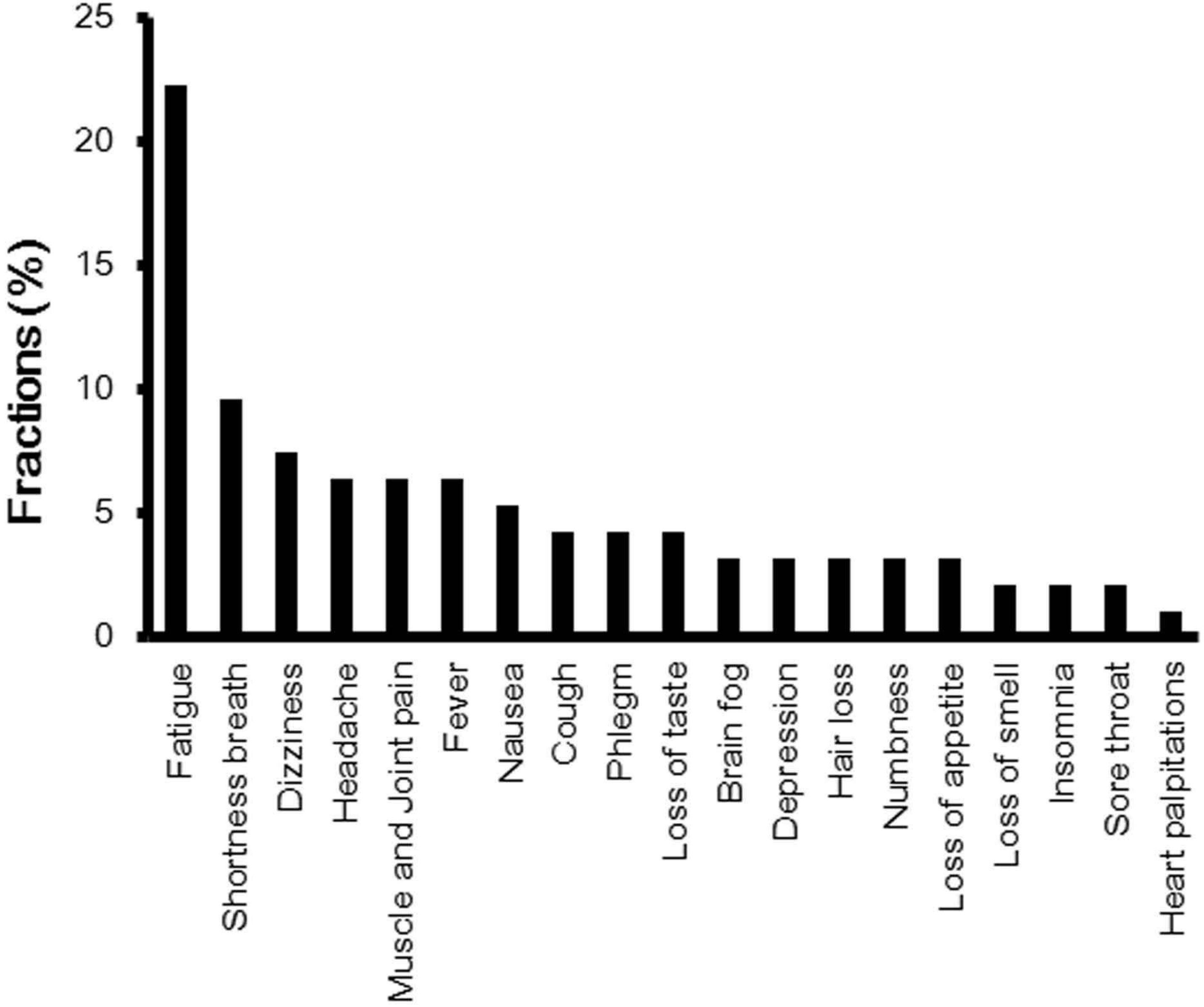

Supplementary Figure 2

LMM

Outcome \ Fixed predictor	IFN a2	IFNb	IFNg	IFNl1	IFNl2/3
IFN a2		n.d.	n.d.	n.d.	n.d.
IFNb	0.49015		n.d.	n.d.	n.d.
IFNg	0.000352	0.00081		n.d.	n.d.
IFNl1	0.8799	0.00727	0.365		n.d.
IFNl2/3	0.0487	0.0385	0.000015	0.7955	

GLMM

Outcome \ Fixed predictor	IFN a2	IFNb	IFNg	IFNl1	IFNl2/3
IFN a2		n.d.	n.d.	n.d.	n.d.
IFNb	6.22E-11		n.d.	n.d.	n.d.
IFNg	<2e-16	2.00E-16		n.d.	n.d.
IFNl1	<2e-16	<2e-16	0.00277		n.d.
IFNl2/3	5.25E-09	0.0127	<2e-16	8.69E-09	

n.d., not determined

Supplementary Figure 3

LMM

Fixed predictor

Outcome		IFN a2	IFNb	IFNg	IFNl1	IFNl2/3	Anti-DNA	Anti-Sm
Outcome	Anti-DNA	0.0414	0.771	0.0655	0.128	0.9497		0.307
Outcome	Anti-Sm	0.78995	0.326	0.788	0.2341	0.4851	0.328	

GLMM

Fixed predictor

Outcome		IFN a2	IFNb	IFNg	IFNl1	IFNl2/3	Anti-DNA	Anti-Sm
Outcome	Anti-DNA	1.34E-08	<2e-16	1.53E-14	<2e-16	0.000241		0.0869
Outcome	Anti-Sm	0.0039	0.0000316	0.372	1.06E-11	0.047	n.c.	

n.c., not calculated

Supplementary Figure 4

LMM

Fixed predictor

		ISG15	PKR	IFIT1	Mx1
Outcome	IFN a2	0.128	0.0824	0.00902	0.215
	IFNb	0.978	0.93	0.612	0.245
	IFNg	0.407	0.453	0.266	0.2554
	IFNl1	0.612	0.361	0.81	0.0597
	IFNl2/3	0.289	0.485	0.9429	0.0236
	Anti-DNA	0.69	0.702	0.07	0.387
	Anti-Sm	0.949	0.261	0.96	0.787

GLMM

Fixed predictor

		ISG15	PKR	IFIT1	Mx1
Outcome	IFN a2	<2e-16	3.97E-14	<2e-16	0.000132
	IFNb	<2e-16	<2e-16	<2e-16	2.00E-16
	IFNg	0.914	0.317	0.033	0.772
	IFNl1	0.143	<2e-16	1.78E-12	1.01E-07
	IFNl2/3	<2e-16	3.63E-12	<2e-16	<2e-16
	Anti-DNA	0.0869	0.614	0.0307	0.313
	Anti-Sm	0.955	0.00101	0.6204	0.196

Supplementary Figure 5

Anti-DNA	Estimate	Std.Error	t value	Pr(>|t|)
(Intercept)	1.139361	0.528937	2.154057	0.038634
Cough	3.449847	1.363269	2.530569	0.016339
Depression	3.294532	1.544262	2.133401	0.040417
Hair_loss	5.183796	1.54857	3.347473	0.002048
loss_smell	3.291772	1.544262	2.131614	0.040575
Muscle_Joint_Pain	3.10033	1.248497	2.483249	0.018275

Anti-Sm	Estimate	Std.Error	t value	Pr(>|t|)
(Intercept)	5.54479	0.937119	5.916845	9.94E-07
Fever	5.739344	2.263359	2.535765	0.01584
Heart_palpitations	23.22975	5.132815	4.525734	6.66E-05
Loss_Taste	7.996171	3.060619	2.612599	0.013152

IFN alpha2	Estimate	Std.Error	t value	Pr(>|t|)
(Intercept)	1536.531	152.1201	10.10078	4.75E-12
Cough	977.2927	476.6527	2.050325	4.77E-02
Sore_throat	-1124.3	542.693	-2.0717	4.55E-02

IFN beta	Estimate	Std.Error	t value	Pr(>|t|)
(Intercept)	3942.182	291.9895	13.50111	1.17E-15
Loss_Taste	2835.818	1011.481	2.803629	8.09E-03
Numbness	2248.485	1011.481	2.222962	3.26E-02

IFN gamma	Estimate	Std.Error	t value	Pr(>|t|)
(Intercept)	1581.781	70.07602	22.57236	7.69E-23
Cough	843.9687	210.2281	4.014539	2.89E-04
Loss_Taste	509.8854	239.3552	2.130246	4.01E-02

IFN lambda1	Estimate	Std.Error	t value	Pr(>|t|)
(Intercept)	1290.528	284.4848	4.536368	5.85E-05
Numbness	3387.139	1025.725	3.302192	0.002133

IFN lambda2/3	Estimate	Std.Error	t value	Pr(>|t|)
(Intercept)	1920.952	157.6278	12.18664	5.86E-14
Cough	958.5845	462.011	2.074809	4.56E-02
Loss_appetite	1419.411	533.4843	2.660642	1.18E-02
Loss_Taste	1337.911	533.4843	2.507873	1.71E-02
Sore_throat	-1104.15	524.948	-2.10335	4.29E-02
